# A new species of *Microcharon* from marine interstitial waters, Shizuoka, Japan (Isopoda, Lepidocharontidae)

**DOI:** 10.3897/zookeys.680.12048

**Published:** 2017-06-14

**Authors:** Jeongho Kim, Wonchoel Lee, Ivana Karanovic

**Affiliations:** 1 Department of Life Science, Hanyang University, Seoul, Korea; 2 Institute for Marine and Antarctic Studies, University of Tasmania, Hobart, Tasmania, 7001, Australia

**Keywords:** interstitial, isopoda, Japan, morphology, taxonomy

## Abstract

A new species of *Microcharon* Karaman, 1934 (Asellota: Lepidocharontidae) is described from Miho-Uchihama beach, Shizuoka, Japan. *Microcharon tanakai* **sp. n.** differs from its congeners by having nine simple, five penicillate setae on antennal article 6; one simple distal seta on article 1 of the mandibular palp and having the apical lobe of male pleopod 1 convex, rounded, armed with seven setae. A key to Asian species of the genus and 16S rRNA of the new species are provided.

## Introduction

Members of the genus *Microcharon* Karaman, 1934, are free-living interstitial isopods. Being highly adapted to the narrow spaces of the interstitial environment, the species possesses an elongated body without visual organs or pigmentation (Coineau 1994, 2000; Wilson and Wägele 1994). When Karaman (1934) described *Microcharon*, it was included in the family Microparasellidae Karaman, 1934. However, following a recent revision (Galassi et al. 2016), the genus is currently a member of the family Lepidocharontidae. Besides *Microcharon*, Lepidocharontidae also includes *Lepidocharon* Galassi & Bruce, 2016 and *Janinella* Albuquerque, Boulanouar & Coineau, 2014. The most prominent diagnostic character of *Microcharon* is the shapes of pereonites 1-7. Pereonites are cylindrical and each is rectangularly shaped in dorsal view whereas, *Lepidocharon* and *Janinella* have trapezoidal pereonites (Galassi et al. 2016).

The genus *Microcharon* is one of the best studied groups of the family Lepidocharontidae and 69 species have been described from all over the world (Boyko et al. 2008). The majority of species are known from Europe, especially from the Mediterranean region (Coineau 1994; Boyko et al. 2008; Galassi et al. 2016). On the other hand, only the following three Asian species have been recorded so far: *M. halophilus* Birstein & Ljovuschkin, 1965; *M. kirghisicus* Jankowskaya, 1964; and *M. raffaellae* Pesce, 1979. They are known from Turkmenistan, Kyrgyzstan, and Iran respectively (Coineau 1994; Boyko et al. 2008; Galassi et al. 2016). Diversity of *Microcharon* in East Asia remains particularly unknown. Only Shimomura et al. (2006) briefly noted a single species of *Microcharon* collected together with an ingolfiellidean amphipod from marine interstitial of Okinawa archipelago.

During a survey of the interstitial fauna of Miho-Uchihama beach (Shizuoka, Japan), a small number of psammolittoral isopods were collected together with other marine interstitial fauna such as, harpacticoid copepods, nematodes and ostracods. The isopod specimens had a typical *Microcharon* body plan, but a unique combination of morphological characters which lends support to the establishment of the new species described herein. Beside its description an identification key to the four Asian species of this genus currently recorded. In addition, a partial sequence of 16S rRNA gene was obtained and this may be useful for the future phylogenetic study studies of *Microcharon* and the family Lepidocharontidae.

## Materials and methods

### Specimen collection and identification

**Figure 1.**
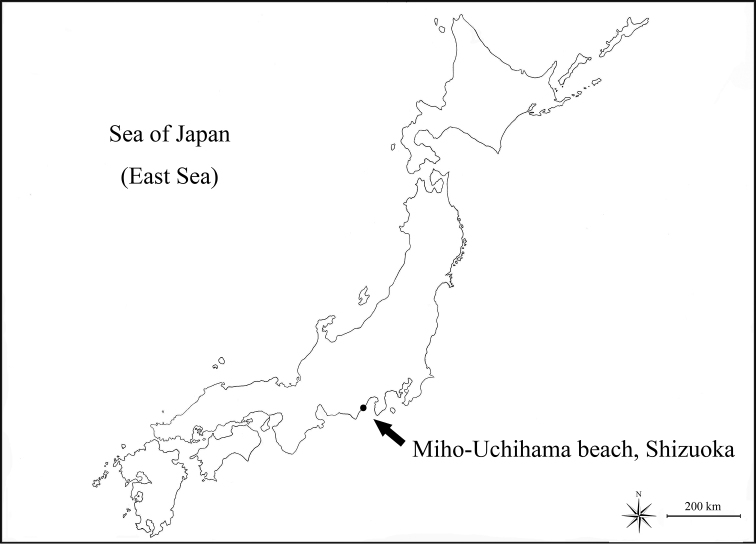
Sampling site. The arrow indicating the type locality of *Microcharon tanakai* sp. n. (Miho-Uchihama beach, Shizuoka, Japan).

Seven female and two male specimens were collected from coarse sand from Miho- Uchihama beach, Shizuoka City, Shizuoka Prefecture, Japan on 7 February 2015 (Fig. 1). Coarse sand samples were washed five times in a bucket with fresh water. The top layer of water was strained through nets of 40 µm mesh size and material was immediately preserved in 99% ethanol. Sorting from sediment sample and dissection of specimens were done under an Olympus SZX 12 dissecting microscope. Dissected appendages were mounted onto glass slides in lactophenol. Line drawings were prepared using a Zeiss Axioskop 50 compound microscope equipped with a camera lucida. All studied material was deposited at the invertebrate collection of the National Institute of Biological Resources (NIBR), Korea. Two female and one male were transferred to isoamyl acetate for 20 minutes and dried in a critical-point dryer Hitachi E-1010. Dried specimens were mounted onto a SEM stub and coated with gold using a sputter coater to a thickness of 15-30 nm. Coated specimens were examined and photographed with a Hitachi S-3400 scanning electron microscope at Chungang University (Seoul). Measurements were done following the method of Riehl and Brandt (2010). All measurements were taken from the dorsal view of line drawings using the distance measurement tools of Adobe Acrobat Professional. The ratios of appendages were given in distal to proximal order, excluding setae. The body ratios were given in anteromedial to posteromedial point order excluding appendages. Whole body length was measured from biggest female specimen. Terminology is largely based on Galassi et al. (2016).

### DNA extraction and amplification

Two females from the type locality sample were identified without dissection under an Olympus SZX 12 dissecting microscope. Before amplification, specimens were transferred into distilled water for 20 minutes to remove ethanol and then macerated with a small glass rod. Whole specimens were used to isolate genomic DNA with the aid of the LaboPassTM Kit (COSMO Co. Ltd., Korea) following the manufacturer’s protocols. 16S rRNA was amplified with polymerase chain reaction (PCR) using PCR premix (BIONEER.Co) in TaKaRa PCR thermal cycler (TaKaRa Bio Inc., Otsu, Shiga, Japan). The primers used were 16sar-L (5`-CWAAYCATAAAGAYATTGGNAC-3`) and 16sar-H (5`-ACTTCAGERTGNCCAAARAAYCA-3`) (Palumbi et al. 1991). The amplification protocol consisted of the initial denaturation at 94°C for 2 min and 35 cycles each consisting of denaturation at 94°C for 50 sec, annealing at 50°C for 50 sec, extension at 72°C for 1 min 20 sec; the final extension was at 72°C for 7 min. Successful amplifications were confirmed by electrophoresis on 1% agarose gel. The PCR products were purified for sequencing reactions, using the Labopass PCR Purification Kit (COSMO Co. Ltd., Korea) following the guidelines provided with the kit. DNA was sequenced on an ABI automatic capillary sequencer (Macrogen, Seoul, Korea) using the same set of primers. All obtained sequences were visualized using Finch TV version1.4.0 (http://www.geospiza.com/Products/finchtv.shtml). Each sequence was checked for the quality of signal and sites with possible low resolution, and corrected by comparing forward and reverse strands.

## Systematics

### Suborder Asellota Latreille, 1802

#### Family Lepidocharontidae Galassi & Bruce, 2016

##### Genus Microcharon

Taxon classificationAnimaliaIsopodaLepidocharontidae

Karaman, 1934

###### Type species.

*Microcharon stygius* (Karaman, 1933)

###### Genus diagnosis

**(modified from Galassi et al. 2016).** Body cylindrical; rostrum weakly developed or absent; antennula 5 or 6 articles; antennal flagellum longer than podomere, pereonites rectangular in dorsal view, with subparallel lateral margins; free pleonite as wide as preonite 7; pereopodal coxal plate hardly discernible, incorporated to sternite body wall; distolateral lobe of male pleopod 1 with folded hyaline lamella running parallel to lateral margin; female operculum as long as pleotelson, with two or four apical setae; well-developed uropods with slender endopod and exopod.

##### Microcharon tanakai sp. n.

Taxon classificationAnimaliaIsopodaLepidocharontidae

http://zoobank.org/638F2AF3-DF77-4293-8077-8E3ECC810F82

Figures 2 , 3 , 4 , 5 , 6 , 7 , 8 , 9 , 10

###### Type locality.

Interstitial water of coarse sand, Miho-Uchihama beach, Shizuoka city, Shizuoka Prefecture, Japan, 35°01'83"N, 138°51'71"E (Fig. 1).

###### Material examined.

Holotype: adult female, (NIBRIV0000787789) completely dissected and mounted in lactophenol on four slides; paratype 1 female (NIBRIV0000787790) dissected on three slides, paratype 2 female (NIBRIV0000787791) dissected on one slide; adult male pleotelson dissected on three slides; 2 females and 1 male used for SEM.

###### Diagnosis.

Antennular article 6 smallest, with one aesthetasc, one penicillate, three simple setae distally; article 1 of mandibular palp with one distal simple seta; lateral lobe of maxillula with eleven robust setae; distal apex of male pleopod 1 convex, round, with three apical, four subapical setae; protopod of male pleopod 2.9 times longer than wide; protopod of uropod 3.1 times longer than wide, with fifteen setae.

###### Description of the female holotype.

**Figure 2.**
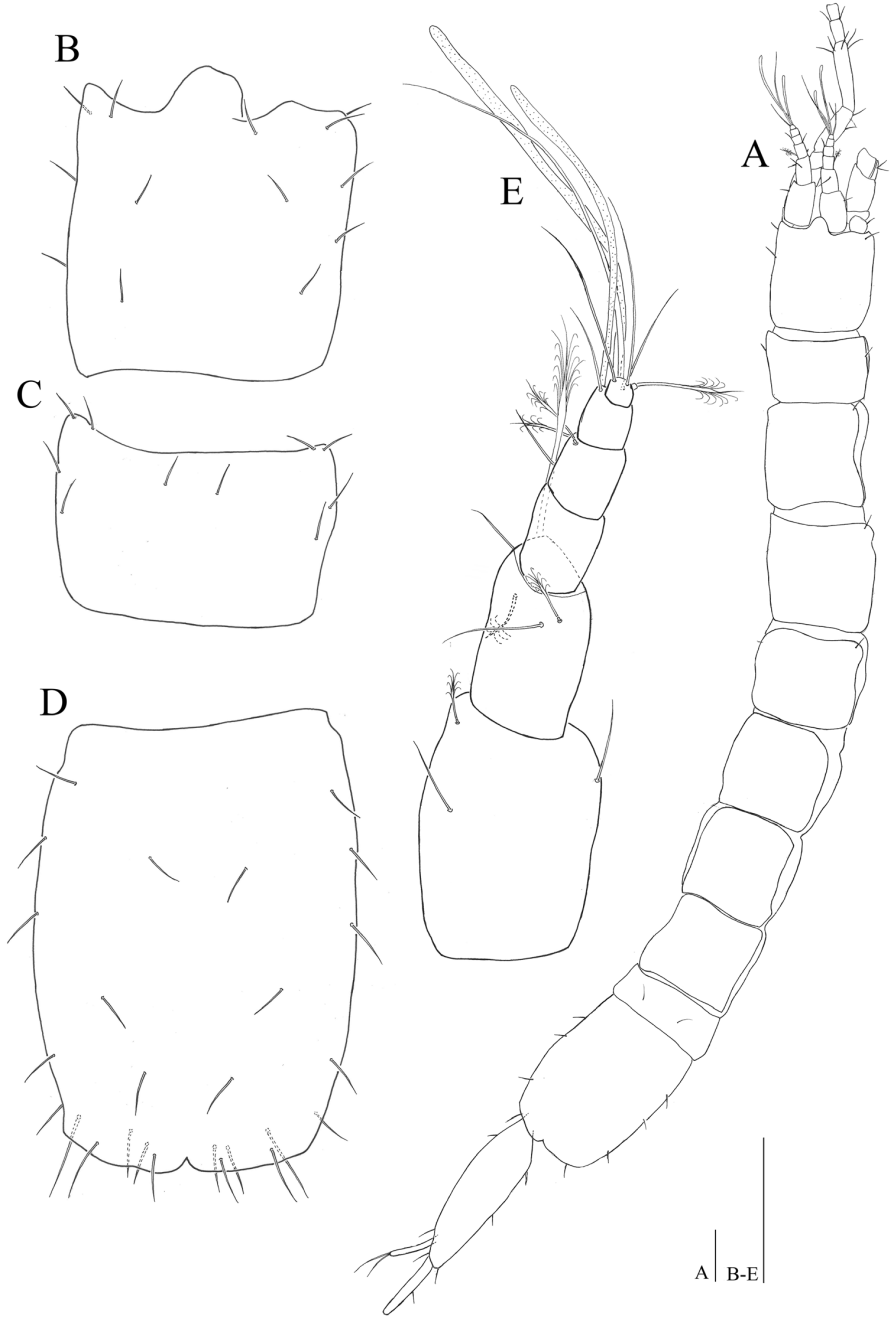
*Microcharon tanakai* sp. n., holotype, female. **A** habitus, dorsal **B** cephalon, dorsal **C** pereonite 1, dorsal **D** pleotelson, dorsal **E** antennula, dorsal. Scale bars 100 µm.

*Body* (Fig. 2A): elongate, slender in whole appearance, total length, 1.95 mm, measured from anteromedial point of cephalon to posteromedial point of pleotelson, body approximately 8.5 times longer than wide, maximum body width in pereonite 3, 0.92 times of maximum width of pleotelson; color of preserved specimens transparent, whole surface of body with many ornamentation looking like lines.

*Cephalon* (Fig. 2B): 1.12 times longer than wide and 0.12 times of whole body; anterior margin with weak rostrum; lateral margins straight, with four pairs of simple setae, three pairs of simple setae along dorsomedial surface.

*Pereon* (Fig. 2A, C): 0.68 times of whole body length, medial margin of tergite convex, pereonites with lateral margin, straight, pereonite 1, 0.62 times longer than wide, four simple setae along lateral margin, two setae on dorsal surface; pereonite 2, 1.12 times longer than wide, with four pairs of setae along lateral margin, two setae on anteromedial edge; pereonite 3, 1.06 times longer than wide, with four pairs of setae along lateral margin, two pairs of setae along dorsal surface; pereonite 4, 1.02 times longer than wide, with nine setae on both lateral and dorsal surface; pereonite 5, 0.99 times longer than wide, with six setae on both lateral and dorsal margin; pereonite 6, 1.13 times longer than wide, with eight simple setae on dorsal margin; pereonite 7, 1.02 times longer than wide, with two setae on both anterolateral corner and three pairs of setae along dorsal surface.

*Pleonite 1* (Fig. 2A): as wide as pereonite 7, 0.38 times longer than wide, with two simple setae on dorsomedial margin.

*Pleotelson* (Fig. 2D): 1.33 times longer than wide, wider than preceding pereonites, with several setae, becoming slightly narrow from basal part to distal end, weak cleft on middle of posterior rim.

*Antennula* (Fig. 2E): 6 articles; article 1 robust, 1.5 times longer than wide, with three setae: two distomedial simple, one penicillate short setae distolaterally; article 2, smaller than 1, distoventral projection, 1.8 times longer than wide, with two simple, three penicillate setae, one of them elongate, stout, reaching distal tip of antennula; article 3, naked, 1.5 times longer than wide; article 4 with one proximolateral simple seta, two penicillate setae distolaterally; article 5, 1.2 times longer than wide with one simple seta, one aesthetasc distomedially; article 6, smallest 0.6 times of article 5, with one aesthetasc, one penicillate, three simple setae on distal end.

**Figure 3.**
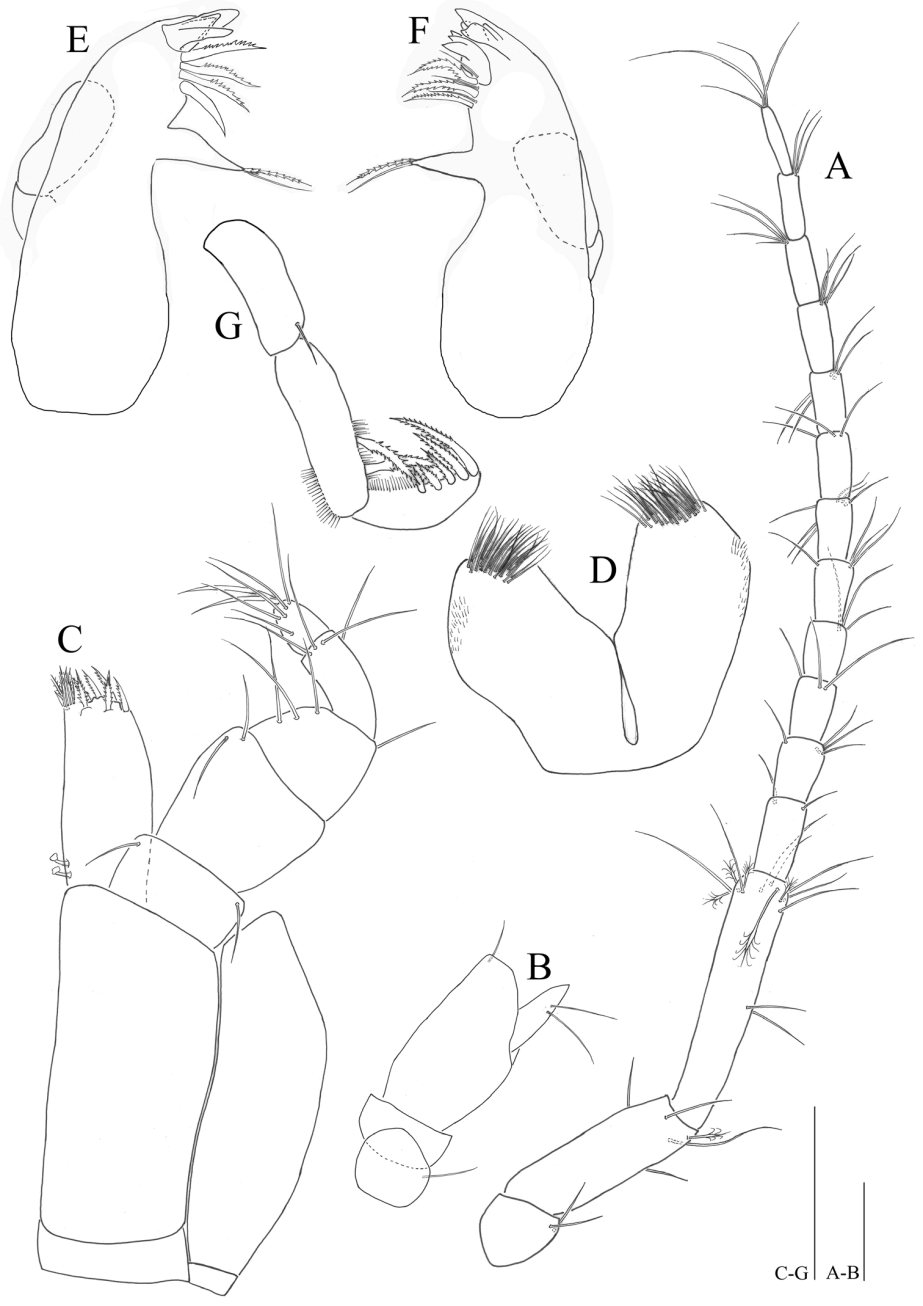
*Microcharon tanakai* sp. n., holotype, female. **A** antennal articles 4-6 with flagellum, dorsal **B** antennal articles 1-3, dorsal **C** maxilliped dorsal **D** paragnaths dorsal **E** left mandible, dorsal **F** right mandible, dorsal **G** mandibular palp, ventral. **A–B** scale bars 100µm, **C–G** scale bars 50 µm.

*Antenna* (Fig. 3A, B): six podomeres, twelve flagellar articles; article 1 globular in dorsal view, with one lateral simple seta; article 2 semicircular, naked; article 3, 1.8 times longer than wide, with one distomedial simple seta, scale thick, robust, with two simple setae laterally (Fig. 8A); article 4, as long as wide, with two simple setae distolaterally; article 5, 3.02 times longer than wide, with three distoventral simple setae, one simple, one penicillate setae distodorsally; article 6, longest, 5.3 times longer than wide, with two simple, five penicillate setae on lateral margin, seven simple setae on distal margin; flagellar articles from 2 to 8 subequal in length, armature of each articles as follows: article 1 with two simple setae distally, article 2 with four simple setae distally, article 3 with three simple setae distally, article 4 with three simple setae distally, article 5 with four simple setae distally, article 6 with six simple setae distally, article 7 with three simple setae distally, article 8 with four simple setae distally, article 9 with four simple setae distally, article 10 with four simple setae distally, article 11 with three simple setae distally, article 12 with four simple setae distally

*Mandible* (Figs 3E–G, 8B): body robust, curved inwardly; *pars incisiva* of both mandibles with four cusps; right mandible (Fig. 3F), *lacinia mobilis*, with five cusps, tapering proximally three pinnate, three simple setae located below *lacinia mobilis*; *pars molaris* of both mandibles with one simple, one pinnate distal setae, slightly longer than *pars molaris*, almost same length as seta on palp; left mandible (Fig. 3E), *pars incisiva* with four cusps, *lacinia mobilis* missing, with three pinnate and one naked robust setae located below *pars incisiva*; palp (Fig. 3G) with three articles, inserted on cuticular projection; article 1 with one simple seta distally (Fig. 8B), article 2 longest, with two pinnate and several hair-like setae on distal margin, article 3 curved outwardly, with five pinnate and with several hair-like setae on lateral margin.

*Paragnaths* (Fig. 3D): deeply incised, with two lobes, each distal part of lobes covered with numerous setae.

**Figure 4.**
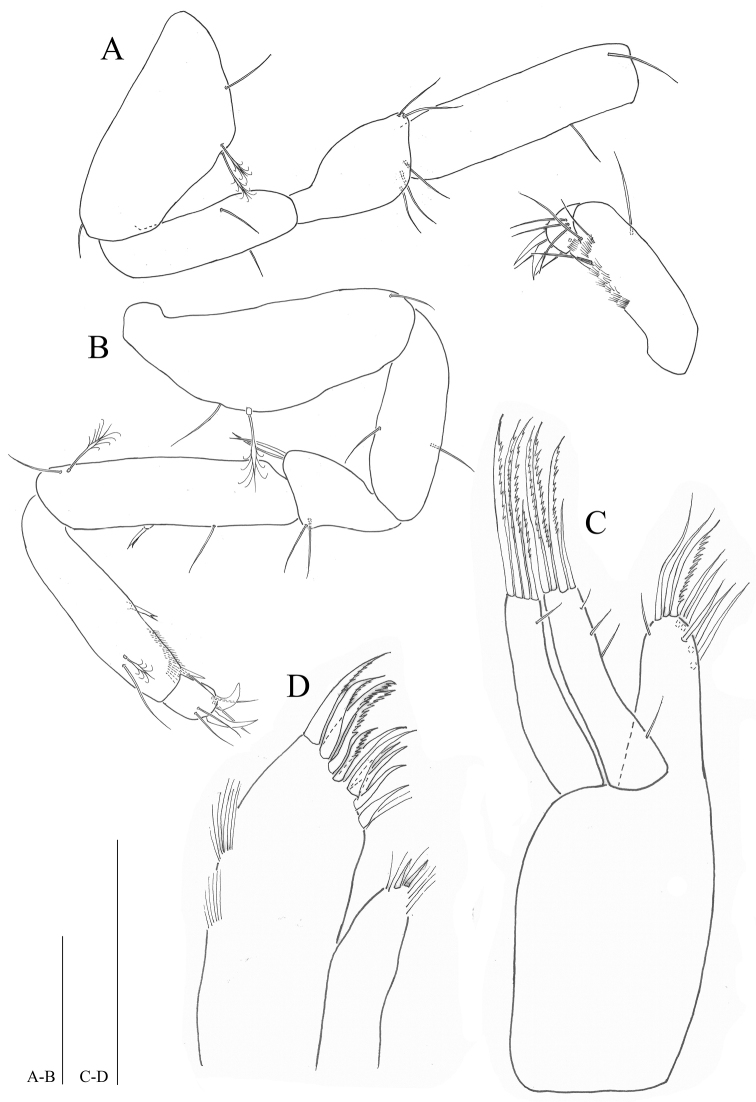
*Microcharon tanakai* sp. n., holotype, female. **A** pereopod 1, lateral **B** pereopod 2, lateral **C** Maxilla, ventral **D** Maxillula, ventral. Scale bars 50µm.

*Maxillula* (Fig. 4D): lateral lobe with eleven strong robust setae on distal edge, some denticulate, some setulose, two spinular rows along outer margin; mesial lobe nearly half as long and wide as outer endite, with three short simple and several hair-like setae on distal end.

*Maxilla* (Fig. 4C): lateral rami slightly shorter than median one, with four pectinate setae on distal end respectively; mesial ramus coalescent with basis, much thicker than others with eight strong setae distally and one of them pectinate.

*Maxilliped* (Fig. 3C): epipodite wide, apically pointed, distal tip reaching distal part of first palp article; basis 2.1 times longer than wide; palp with 5 articles; article 1, 0.5 times longer than wide, with two simple setae on both distal corners; article 2, 1.7 times longer than article 1, with two simple setae on distomedial corner; article 3 tapering distally, with three simple setae along medial margin, one seta on distolateral corner; article 4 curved inwardly, with four simple setae distally; article 5 with five simple setae, 2 claws distally; endite 2.4 times longer than wide, with five apical pinnate setae, numerous spinules on distomedial corner.

**Figure 5.**
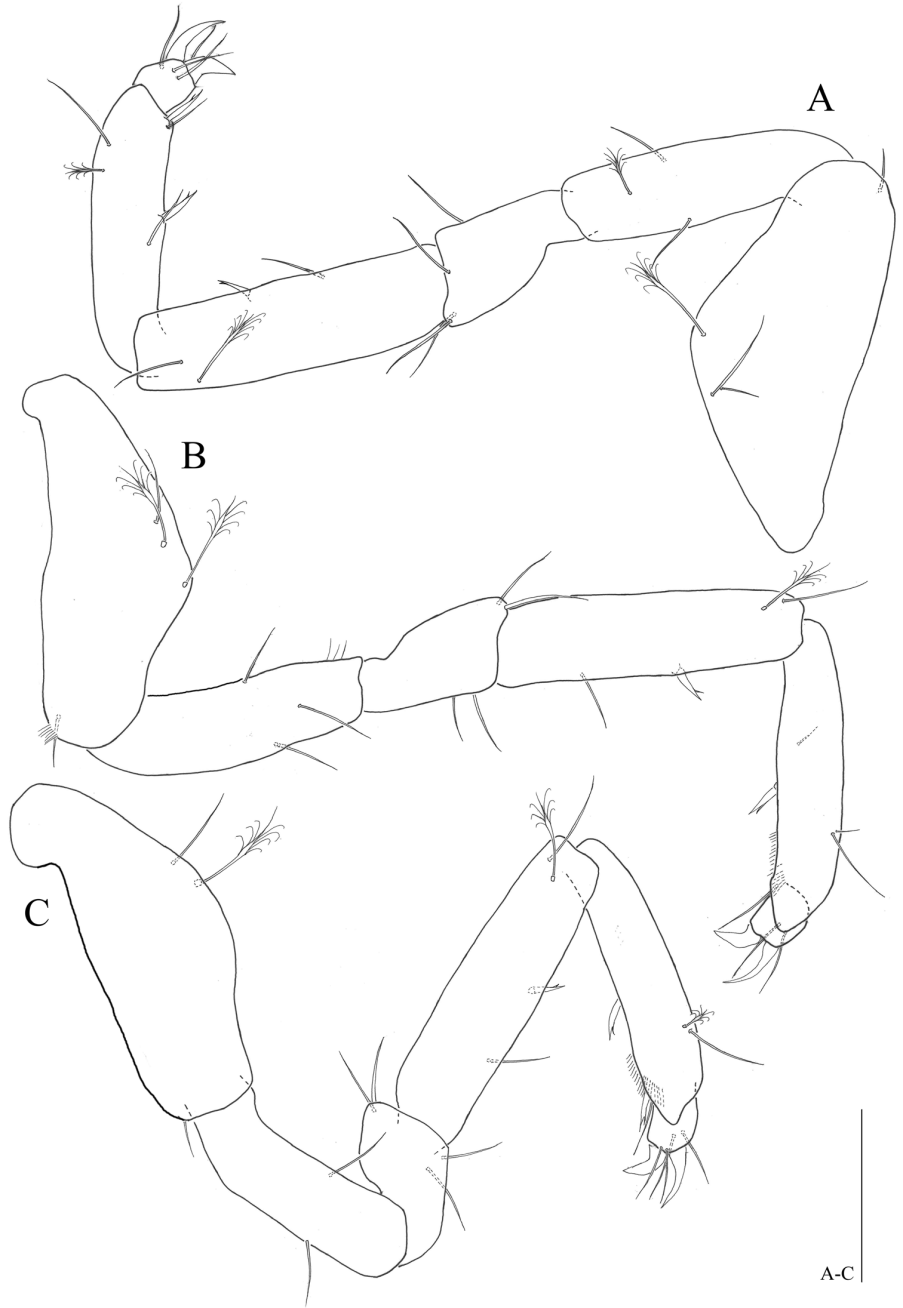
*Microcharon tanakai* sp. n., holotype, female. **A** pereopod 3, lateral **B** pereopod 4, lateral **C** pereopod 5, lateral. Scale bars 50µm.

*Pereopods* 1–4 (Figs 4A, B, 5A, B): inserted on pereon anterolaterally (Fig. 8C); coxal plate minute, hardly discernible (Fig. 8D); anteromedial margin of basis protruded, with one small seta on distal corner, pereopod 1 and 4 with two penicillate, one simple setae (vs pereopod 2, with one penicillate, one simple setae; pereopod 3 with one penicillate, two simple setae); ischium, pereopod 1 and 2 with two simple setae on both anteroposterior margin (vs pereopod 3 with two simple, one penicillate setae; pereopod 4 with three simple setae); merus, shortest article, broaden distally, distinctly shorter than ischium, pereopod 2-4 with four simple setae on both side of distal corner (vs pereopod 1 with five simple setae); carpus, longer than ischium, pereopods 2-4 with one bifid, two simple, one penicillate setae (Fig. 8E, F), (vs pereopod 1 with two simple setae); propodus, slightly shorter than carpus, pereopod 1 with four simple setae (vs pereopod 2 with two bifid, two simple, one penicillate setae; pereopod 3 with two bifid, three simple, one penicillate setae; pereopod 4 with one bifid, four simple setae); pereopod 4 with distal sclerite covering proximal part of dactylus, dactylus with two claws (Fig. 8E), two pairs of setae on both dorsal and ventral margin (vs pereopod 1 with three simple, two simple setae on both dorsoventral margin respectively).

**Figure 6.**
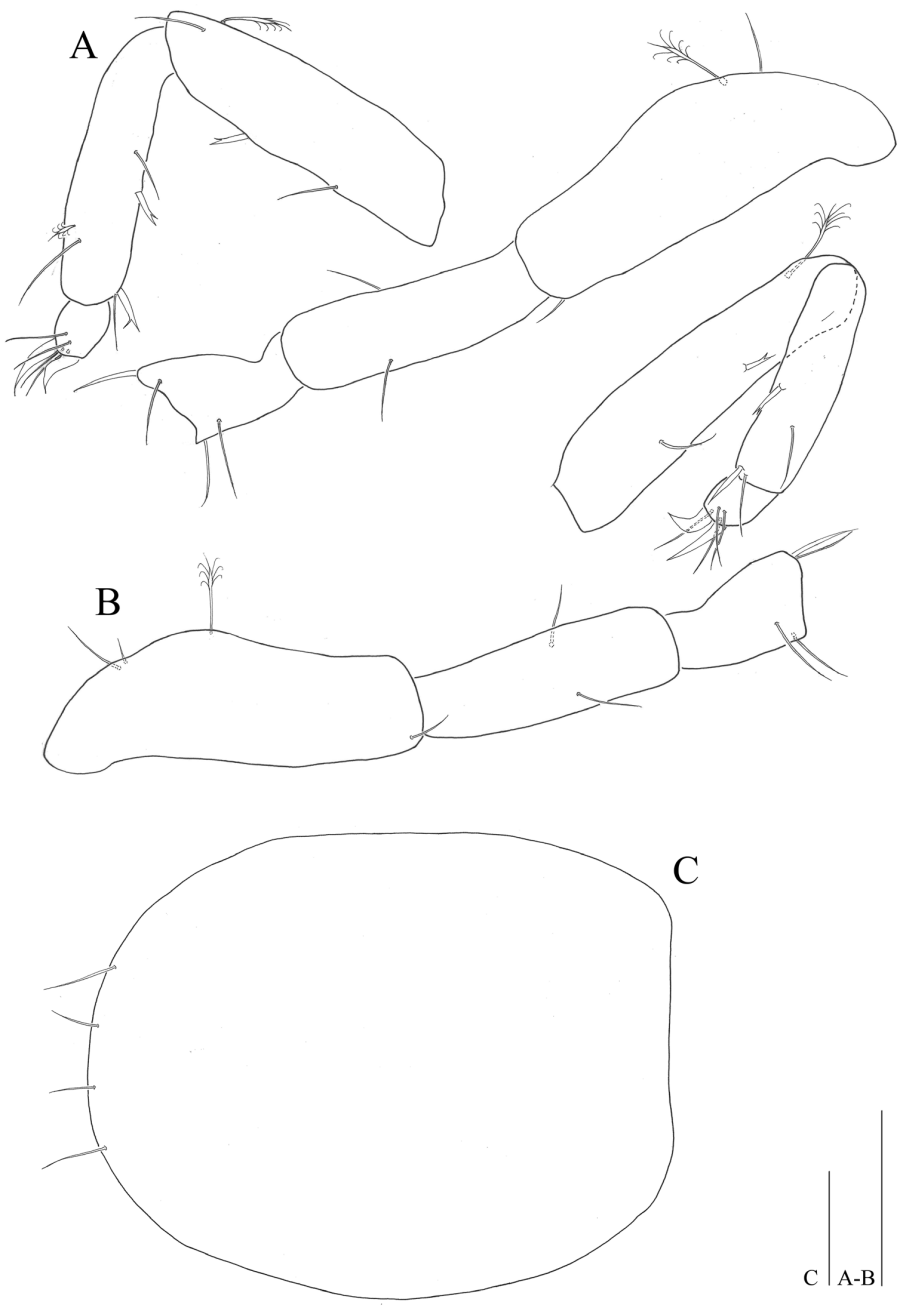
*Microcharon tanakai* sp. n., holotype, female. **A** pereopod 6, lateral **B** pereopod 7, lateral **C** operculum, dorsal. Scale bars 50µm.

*Pereopods* 5–7 (Figs 5C, 6A, B): inserted on pereon posterolaterally, subequal in whole appearance with preceding ones, with small differences in chaetotaxy; coxal plate minute, hardly discernable; basis, with one small seta on distal corner, pereopod 5 with one simple, one penicillate, setae on anteromedial margin (vs pereopod 7 with two simple, one penicillate setae); ischium, with two simple setae on both anteroposterior margin; merus, with four simple setae on both side of distal corner; carpus, with one bifid, two simple, one penicillate setae (vs pereopod 7 with one bifid, one simple, one penicillate setae); propodus, pereopod 5 with two bifid, two simple, one penicillate setae (vs pereopod 6 with two bifid, thre simple, one penicillate setae; pereopod 7 with two bifid, two simple setae), with sclerite on distal margin, covering proximal part of dactylus (Fig. 8F); dactylus with two claws, two pairs of setae on both dorsal, ventral margins.

*Female operculum* (Fig. 6C): 1.2 times longer than wide, with no ornamentation on dorsal surface, with four setae on distal margin.

**Figure 7.**
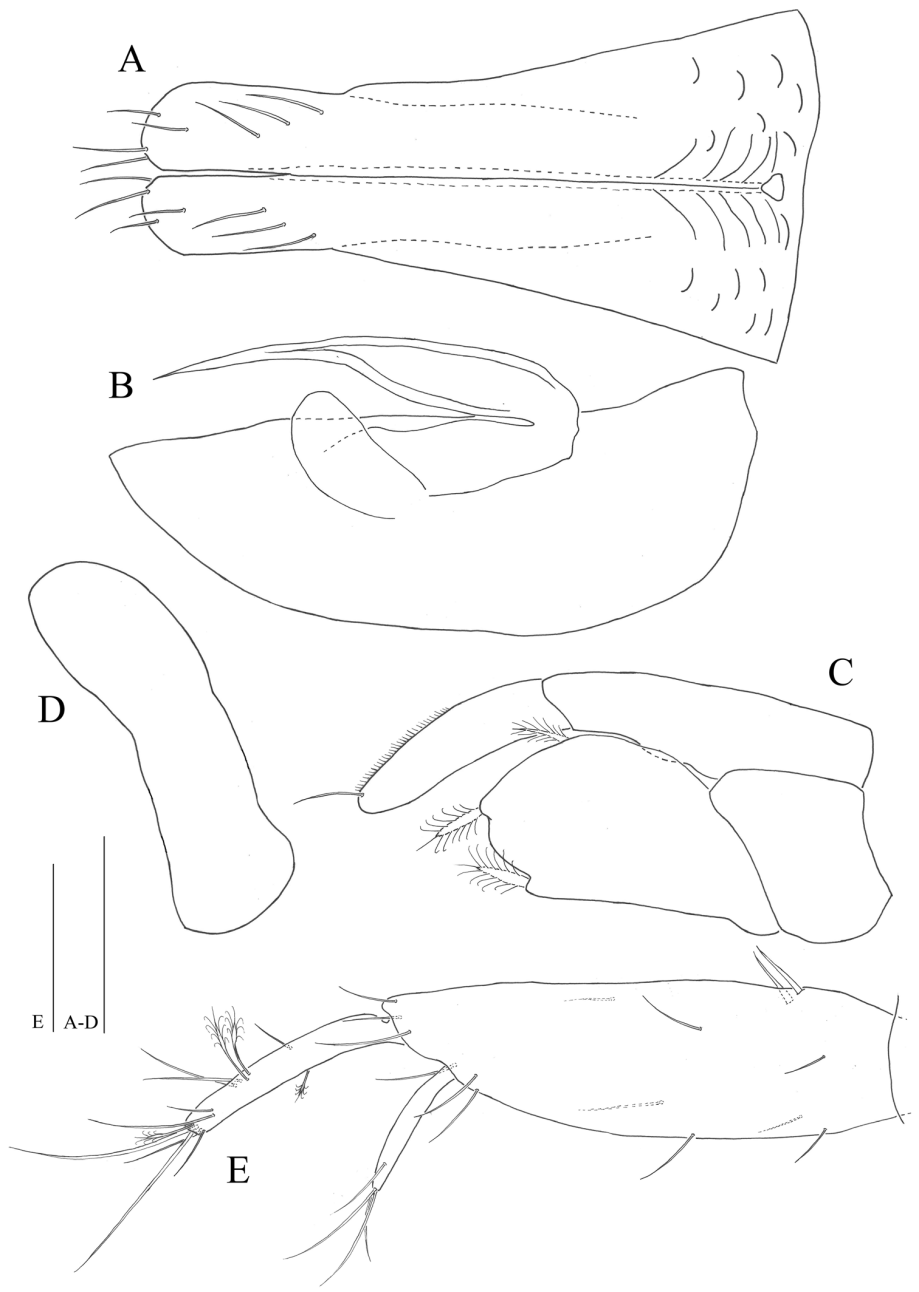
*Microcharon tanakai* sp. n., male. **A** pleopod 1, dorsal **B** pleopod 2, dorsal **C** pleopod 3, dorsal **D** pleopod 4, dorsal **E** female right uropod, dorsal. Scale bars 50µm.

*Uropods* (Fig. 7E): protopod robust, slightly longer than pleotelson, length 3.1 times longer than wide, with thirteen simple and two robust setae proximomedially; endopod 0.5 times longer than protopod, 1.7 times longer than exopod, with nine simple and four penicillate setae; exopod 0.6 times longer than endopod, with four simple setae distally.

**Figure 8.**
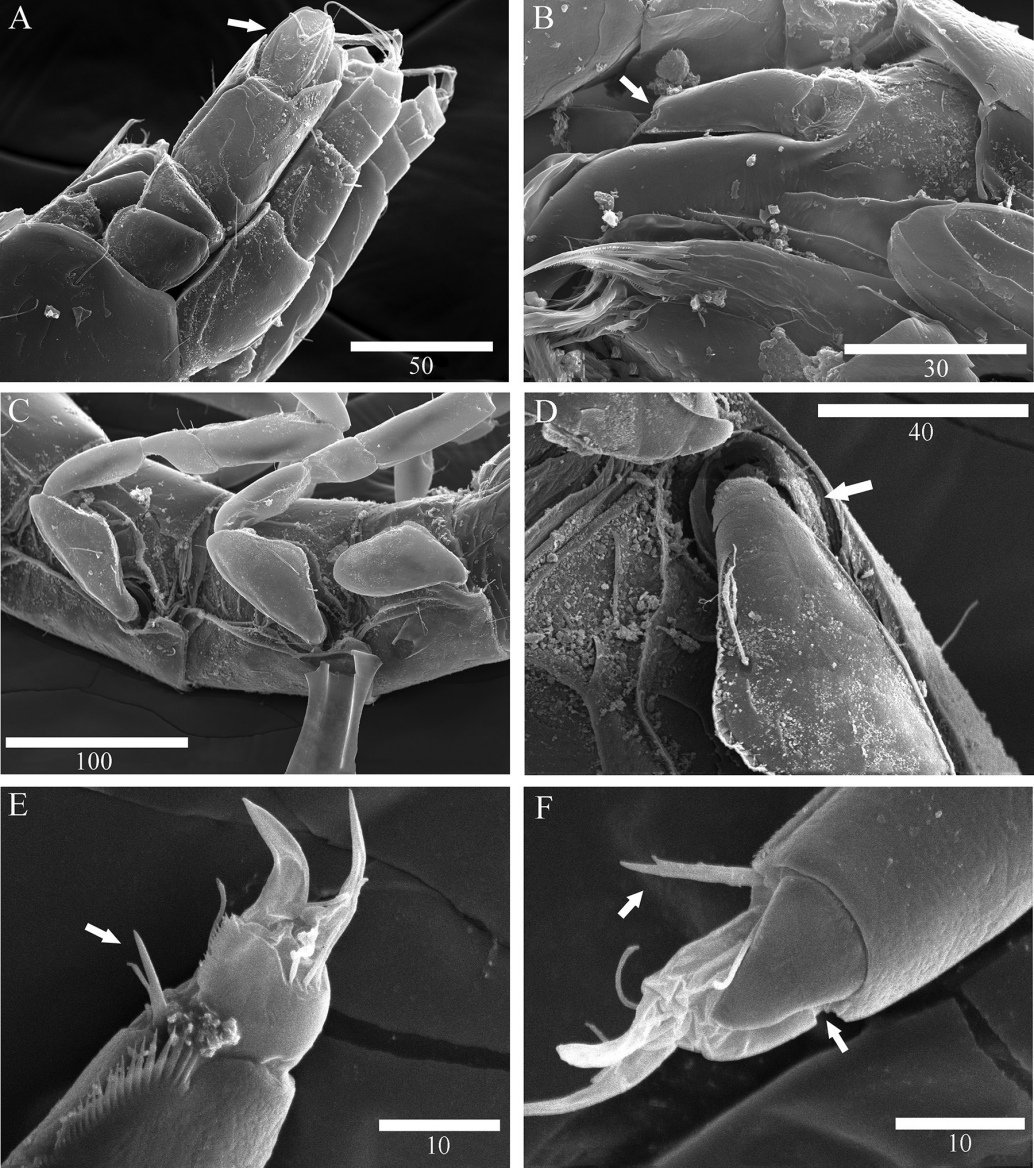
Scanning electron microscope images of *Microcharon tanakai* sp. n., **A** female antennal scale, lateral, **B** female mandibular palp, lateral **C** female pereopod 1-3, lateral, **D** female coxal plate of perepod 2, ventral **E** female bifurcate seta of pereopod 4, lateral, **F** female articular plate of pereopod 5, lateral. Scale bar unit µm.

###### Description of male.

**Figure 9.**
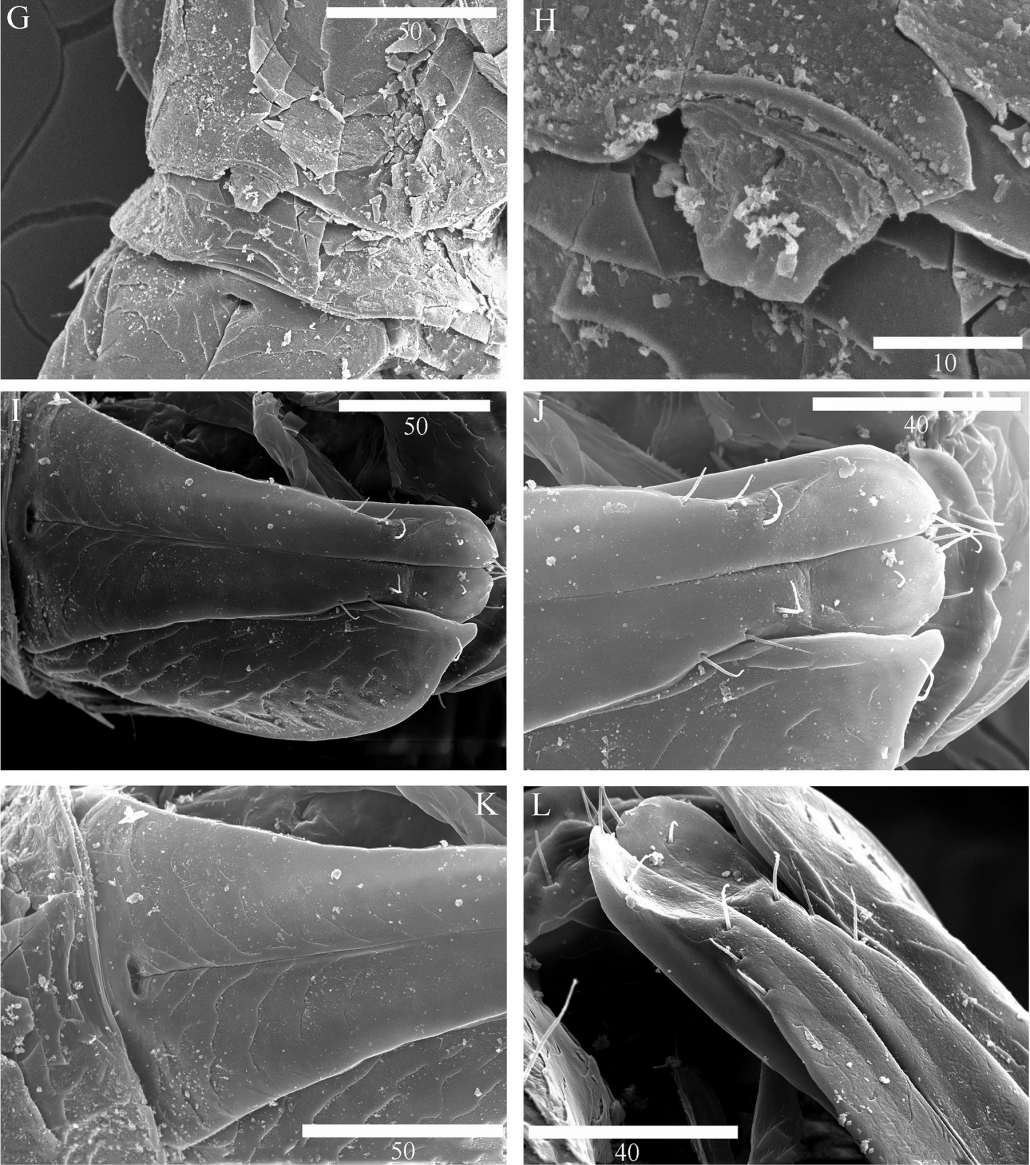
Scanning electron microscope images of *Microcharon tanakai* sp. n., **G** pereonite 7 and free pleonite, ventral, paratype male **H** penial papillae, ventral, **I** pleopod 1, dorsal, **J** distal part of male pleopod 1, dorsal **K** proximal part of male pleopod 1, dorsal **L** distolateral view of male pleopod 1. Scale bar unit µm.

*Penial papillae* (Fig. 9G, H): located at posteromedial margin of pereonite 7 in ventral view, coalescent, proximal margin round, tapering distally, but distal margin straight, with proximomedial opening channel.

**Figure 10.**
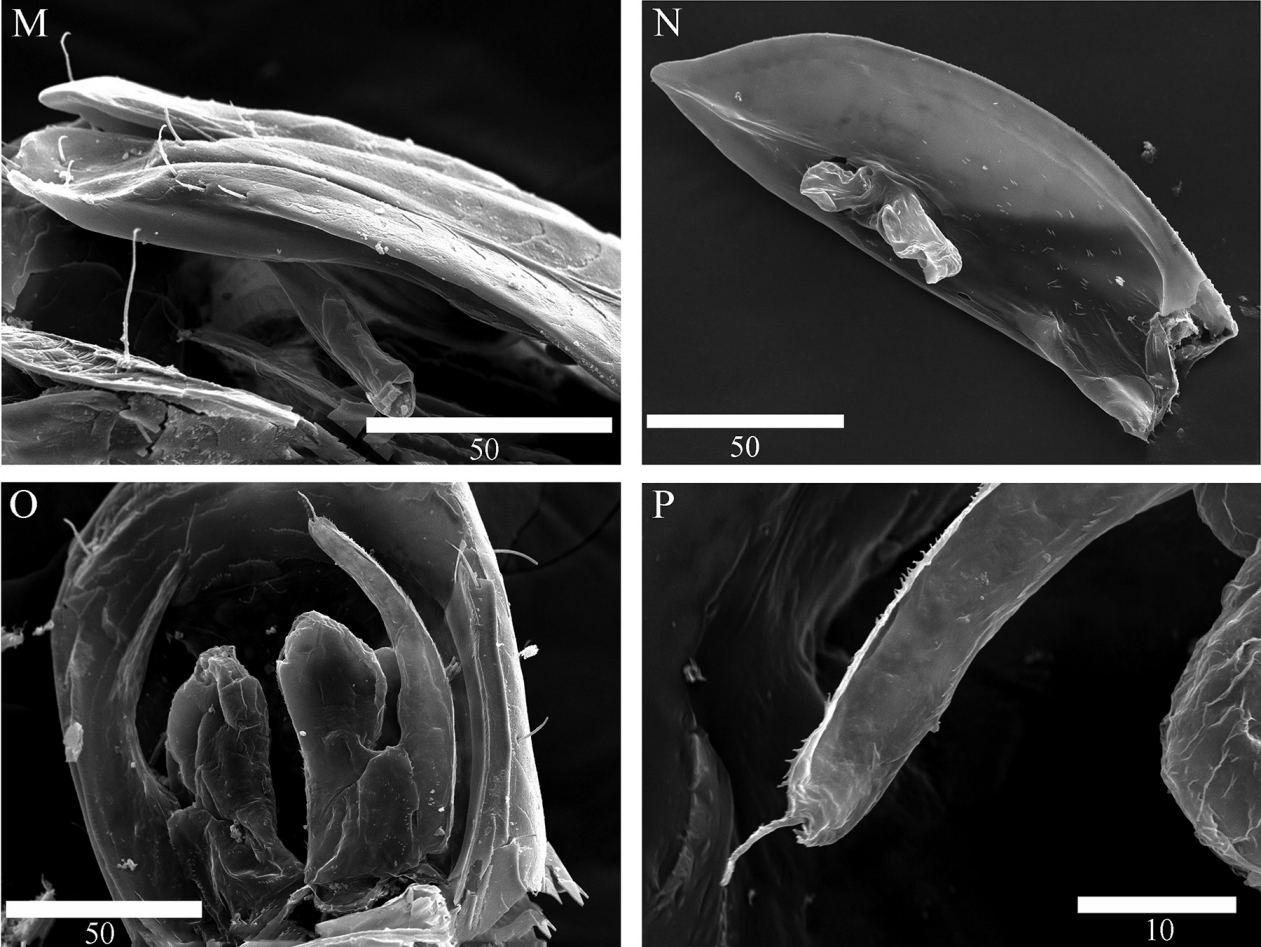
Scanning electron microscope images of *Microcharon tanakai* sp. n., **M** lateral view of male pleopod 1 **N** protopod of male pleopod 2, ventral **O** pleopod 3, dorsal, female **J** distal tip of pleopod 3 exopod. Scale bar unit µm.

*Pleopod 1* (Figs 7A, 9I–L, 10M): elongate, total length almost reaching posterior margin of pleotelson, composed of two coalescent halves, proximal part enlarged, gradually tapering at its distal part, approximately 1.9 times longer than maximum wide (measured at widest section of proximal part); separated in half by medial sperm tube running from triangular opening on proximal part of medial groove, distolateral edge of hyaline lamella straight, without ornamentations; parallel to lateral margin of pleopod, in lateral view distal end of lobes convex, rounded at distal apex, each with three simple setae distally, four subapical setae on ventral margin.

*Pleopod 2* (Figs 7B, 10N): protopod elongate, robust, 2.9 times longer than wide; appendix masculina, curved, tapering distally, tip nearly reaching to protopod apex, with no armature; exopod rounded apically, broad.

*Pleopod 3* (Figs 7C, 10O, P): no sexual dimorphism, endopod two-articulated, second article suboval, with ornamentation like turtle shell shape, and with one apical, one mesial, one lateral plumose setae; exopod two-articulated, clearly longer than endopod, reaching far beyond tip of endopod, with one simple seta distally.

*Pleopod 4* (Fig. 7D): rudimentary, uniramous, no ornamentation, distal margin rounded.

###### Etymology.

The species is named in honor of the collector, Dr. Hayato Tanaka, to express our appreciation for his support in this study.

### Key to Asian species of *Microcharon*

**Table:** 

1	Endopod of pleopod 3 with three plumose setae; article 1 of mandibular palp with one single seta distally	***M. tanakai* sp. n.**
–	Endopod of pleopod 3 naked; article 1 of mandibular palp naked	**2**
2	Antennula consist of 6 articles	***M. halophilus***
–	Antennula consist of 5 articles	**3**
3	Female operculum with 4 distal simple setae; distal part of male pleopod 1, rounded	***M. raffaellae***
–	Female operculum with 2 distal simple setae; distal part of male pleopod 1, straight	***M. kirghisicus***

### DNA amplification

Partial 16S rRNA sequence was obtained only from a single female. The final length of trimmed sequence was 493 base pairs (GenBank accession number KY498031) and BLAST (Altschul et al. 1990) analysis of the GenBank data base revealed that the obtained sequence was isopod in origin and was not contaminated. *Chelator rugosus* (GenBank accession number KJ578668.1, Desmosomatidae) was the most similar sequence to *M. tanakai* resulted by Megablast optimization with the 74% identity, 1e-36 E-value, 92% of query cover, 165 of total and max score. On the other hand, when Blastn optimization was chosen, *Betamorpha fusiformis* (GenBank accession number EF116503.1, Munnopsidae) was the most similar sequence to that of our new species with 73% identity, 4e-67 E-value, 100% of query cover, and 266 of total and max score.

## Discussion

*Microcharon tanakai* sp. n. is identified as a member of the genus *Microcharon* based on the combination of the following characters: 1) body slender, elongate and all somites subequal in width, 2) pereopods 1-4 inserted anterolaterally, pereopods 5-7 inserted posterolaterally 3) coxal plates indiscernible in dorsal view, 4) pleotelson longer than wide, longer than any pereonite, 5) antennal article three with scale laterally 6) antennal flagellum longer than podomeres 7) maxillipedal palps composed of five articles, which are broader than the endite, 8) pereopod 1 leg-like, 9) uropods with long, broad protopod, both endopod and expod shorter than protopod in all other marine species of *Microcharon* and exopod inserted subapically (Wilson and Wägele 1994; Albuquerque et al. 2014; Galassi et al. 2016). The new species can be clearly distinguished from the three Asian species. Like *M. halophilus* described from the Kaptar-Khana cave, Turkmenistan (Birstein and Ljovuschkin 1965), it shows six articles of antennula. However, their differences include: a weak rostrum on the anterior margin of cephalon in the new species; larger length/width ratio of article 6 of the antennula (1.7 vs 1); setal formula of the antennula (article 1 with two simple, one penicillate setae vs one simple seta); the presence of one simple distal seta on the article 1 of the mandibular palp; only a simple setae along the medial margin (vs with combination of bifid, simple setae) on the carpus and propodus of pereopod; the female operculum with four setae distally (vs two distal setae in *M. halophilus*); and the endopod of the pleopod 3 with three penicillate setae (vs naked in *M. halophilus*). The other Asian species, *M. kirghisicus* and *M. raffaellae* described from Kyrgyzstan (Jankowskaya 1964) and Iran (Pesce 1979) can be easily distinguished from *M. tanakai* by the number of articles on the antennula (5 vs 6); both the carpus and propodus of the pereopod with only one simple seta along the medial margin and a difference in the setal formula of the endopod of pleopod 3 (naked vs with three penicillate setae).

We were able to amplify 16S rRNA partial sequence of *M. tanakai*. To date, 228 16S rRNA sequences of the suborder Janiroidea G.O. Sars, 1897 have been deposited on GenBank belonging to the following 7 families: Desmosomatidae G.O. Sars, 1897, Macrostylidae Hansen, 1916, Joeropsididae Nordenstam, 1933, Janiridae G.O. Sars, 1897, Munnopsidae Lilljeborg, 1864, Haploniscidae Hansen, 1916 and Acanthaspidiidae Menzies, 1962. Although this molecular marker has not been commonly used for barcoding (Hebert et al. 2003; Costa et al. 2007), the 16S rDNA has been proven a valid marker for distinguishing morphologically similar species of families Serolidae Dana, 1852, Chaetiliidae Dana, 1849 and Munnopsidae Lilljeborg, 1864 (Held 2000; Held and Wägele 2005; Raupach et al. 2007).

## Supplementary Material

XML Treatment for Microcharon

XML Treatment for Microcharon tanakai
