# Corrigenda: Kim J, Lee W, Karanovic I (2017) A new species of *Microcharon* from marine interstitial waters, Shizuoka, Japan (Isopoda, Lepidocharontidae). ZooKeys 680: 13–31. https://doi.org/10.3897/zookeys.680.12048

**DOI:** 10.3897/zookeys.683.14669

**Published:** 2017-07-10

**Authors:** Jeongho Kim, Wonchoel Lee, Ivana Karanovic

**Affiliations:** 1 Department of Life Science, Hanyang University, Seoul, Korea; 2 Institute for Marine and Antarctic Studies, University of Tasmania, Hobart, Tasmania, 7001, Australia Corresponding

After our manuscript has been published, we have noticed in our acknowledgment section that the name and the code of our funding program were incorrect. Here, we provide the first sentence of the modified acknowledgment using the correct name and code.

“This study was supported by the BK21 Plus Program **(Eco-Bio Fusion Research Team, 22A20130012352)** funded by the Ministry of Education (MOE, Korea)”.

